# Manufacturing conditioned roughness and wear of biomedical oxide ceramics for all-ceramic knee implants

**DOI:** 10.1186/1475-925X-12-84

**Published:** 2013-08-29

**Authors:** Anke Turger, Jens Köhler, Berend Denkena, Tomas A Correa, Christoph Becher, Christof Hurschler

**Affiliations:** 1 Institute of Production Engineering and Machine Tools (IFW), Gottfried Wilhelm Leibniz Universität Hannover, An der Universität 2, 30823 Garbsen, Germany; 2 Laboratory for Biomechanics and Biomaterials (LBB), Department of Orthopaedic Surgery, Hannover Medical School, Anna-von-Borries-Straße 1-7, 30628 Hannover, Germany

## Abstract

**Background:**

Ceramic materials are used in a growing proportion of hip joint prostheses due to their wear resistance and biocompatibility properties. However, ceramics have not been applied successfully in total knee joint endoprostheses to date. One reason for this is that with strict surface quality requirements, there are significant challenges with regard to machining. High-toughness bioceramics can only be machined by grinding and polishing processes. The aim of this study was to develop an automated process chain for the manufacturing of an all-ceramic knee implant.

**Methods:**

A five-axis machining process was developed for all-ceramic implant components. These components were used in an investigation of the influence of surface conformity on wear behavior under simplified knee joint motion.

**Results:**

The implant components showed considerably reduced wear compared to conventional material combinations. Contact area resulting from a variety of component surface shapes, with a variety of levels of surface conformity, greatly influenced wear rate.

**Conclusions:**

It is possible to realize an all-ceramic knee endoprosthesis device, with a precise and affordable manufacturing process. The shape accuracy of the component surfaces, as specified by the design and achieved during the manufacturing process, has a substantial influence on the wear behavior of the prosthesis. This result, if corroborated by results with a greater sample size, is likely to influence the design parameters of such devices.

## Background

Medical engineering is an important area of technological advancement in the 21st century. The development and manufacturing of medical implants that replace failed body or organ functions is of great importance for an aging population. The number of implants/prostheses continues to increase, which in Germany, led to a total cost increase from 450 million Euro to 1.1 billion Euro from 1996 to 2004 (German Institute for Economic Research, DIW Berlin) [1]. However, currently available implant technology can be improved in areas including biocompatibility, functionality, biointegration, and survivability.

More than five million individuals currently suffer from osteoarthritis in Germany, and in 2008, approximately 170,000 of these were provided with knee endoprostheses. The complication rate of current knee implants is approximately 25% within 20 years. Infection, wear and breakaway are common reasons for revision surgery [2-5], but the major cause of implant failure is implant loosening, often itself related to wear-induced osteolysis. Most knee joint replacements presently involve the articulation of a cobalt-chromium-molybdenum alloy and ultra-high-molecular-weight polyethylene (hereafter denoted CoCr-PE).

A large amount of research and development related to orthopaedic implants currently relates to wear reduction and the prevention of foreign-body reactions through the use of coatings or high-strength materials [4]. At present, wear-resistant, all-ceramic tribological pairings are being used in hip arthroplasties [6,7]. However, these successful tribological pairings are not easily transferable to knee arthroplasties for a variety of design and manufacturing reasons. The complex geometry, surface quality requirements, and typical loading patterns of a knee joint replacement present a genuine challenge when considering the mechanical properties of ceramic materials.

Several studies are presently investigating the possibility of using a high-strength ceramic material for the femoral component of a total knee replacement. Two manufacturers – Kyocera (Japan) and CeramTec (Germany) – have developed such a component as an alternative for patients with metal allergies [6,7]. However, the implant component, which is vulnerable to wear – the polyethylene inlay – remains present. Tibial and femoral components made of ceramic in a hard-hard-pairing may reduce wear and increase implant longevity. As known from hip replacements, ceramic-on-ceramic pairings have vastly different surface requirements to ceramic-on-polyethylene. Therefore, the machining technology required for ceramic-on-ceramic knee prostheses has not been developed to date.

The primary aims of this study were the identification of design and manufacturing requirements of an all-ceramic knee implant, the translation of these requirements into a design, and the realization of this design by an economical, automated manufacturing and machining process. The investigation of the influence of surface machining on the wear behavior of an all-ceramic knee implant was the final aim of this study, which involved answering the following questions:

1. How constant is the machining result, and how do roughness deviations from the production process influence wear behavior?

2. To what extent does the contact geometry of the articulating surfaces of the femoral and tibial components influence wear behavior?

Furthermore, we aimed to determine the extent to which surface roughness influences wear behavior. As such, we performed a pre-investigation regarding this relationship, with a small sample size.

## Methods

### Manufacturing techniques

Ceramic implants originate as sintered components, and the manufacturing process chain for ceramic hip implant components is well-established. Due to geometrical distortions and shape deviations, a green body is manufactured slightly larger than the final product, and is then ground and polished after the sintering and hipping processes. There are up to 60 individual machining steps for even the relatively simple geometry of a ceramic hip replacement. Diamond tools are used in the grinding process, and subsequent polishing is often performed using a free-abrasive grinding machine. Machining accuracy can be specified to shape deviations of < 2 μm and surface roughness values (Ra) of < 20 nm.

In contrast to hip replacements, knee implant components have complex, partly free-form surfaces. Free-form surfaces are industrially milled by machines with five or more axes [8-10]. Such milling processes can only be carried out on ceramic components in a green- or white-body state. Sintering and high-isostatic pressing (HIP) follow this, and the final steps involve grinding and polishing.

**Figure 1:**
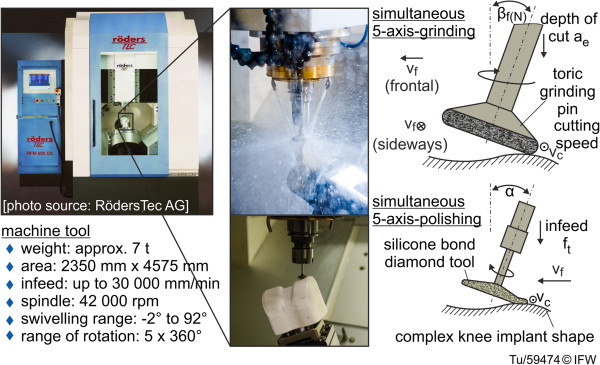
5-axis-machine tool and tool designs for grinding and polishing.

The finishing of metallic knee implant components is usually performed using belt grinding, polishing cloths and free-abrasive grinding processes. Polishing processes result in a smooth surface, and typically account for 10–15% of the total manufacturing cost [11]. For the finishing of complex-shaped ceramic components, a two-step machining process was developed, with both steps able to be performed using the same multi-axis machining center. The 5-axis grinding process generates a macro geometry with a precise surface topography, leading to a reduction in polishing effort. Toric diamond grinding pins are used in this procedure (Figure 1, top) [12-14].

The polishing process employs resilient silicone or polyurethane bond diamond tools which level roughness peaks (Figure 1, bottom). The dimension of material removal during this polishing step is less than 1 μm. The combination of the grinding and polishing steps ensures the requirements regarding shape accuracy and surface quality of the articulating surfaces are met. Previous work by the authors has described in detail the grinding process with toric tools [12-14] and the polishing process with resilient tools [15-20].

**Figure 2:**
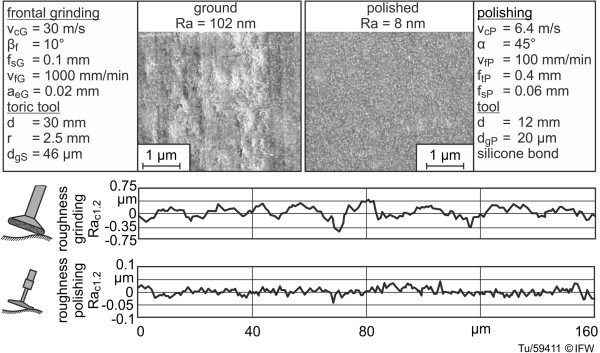
SEM photographs of ground and polished surfaces of simplified components.

For verification of the two-step machining process, implant samples of a zirconia-toughened alumina (ZTA) bioceramic were machined with a galvanic tool by means of frontal grinding, and their topographies were analyzed (e.g., Figure 2, left). A ground surface with a roughness (Ra) of approximately 100 nm was achieved. Following this, the surface was polished with resilient silicone bond diamond tools (Figure 2, right). After polishing, the surface had a roughness (Ra) of 8 nm.

### Surface shape measurement

A coordinate measurement machine (CMM) system (Leitz PMM 866, Hexagon Metrology AG, Wetzlar, Germany) was used for two purposes: assessment of shape accuracy, and measurement of the radii of curvature in both the sagittal and frontal planes. Due to the very short measurement length in the frontal plane, the radius calculation is considerably less accurate than that of the sagittal plane radius. A circle segment of greater than 180° is needed for precise radius measurement, and in industrial measurement, a segment of at least 90° is used [21-23]. Due to the geometry of the samples, only about 4.5% (16,2°) of a full circle was able to be used for measurement of the frontal plane radius for both counterbodies and base plates. For this reason, frontal plane radii were measured three times at three different positions, and the average of these was used in subsequent analysis.

### Wear testing

**Figure 3:**
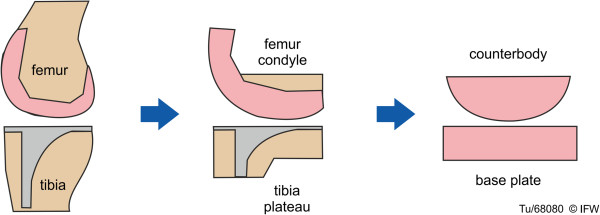
**Development of simplified implant geometry **[24]**,**[25]**.**

In order to analyze the wear behavior of ceramic knee implant components, a wear simulator was developed [24,25] for components with simplified geometries (Figure 3). This machine was intended to be more representative of physiological loading and motions than a pin-on-disk or ring-on-disk tribometer, but at the same time avoiding the complexity of a commercial-grade wear testing device. The surface geometry of the simplified tibial components was planar, and that of the simplified femoral components was semi-cylindrical, with a sagittal-plane radius of 32 mm. The counterbody represents only one of the two articulating surfaces of a knee prosthesis’ femoral component (e.g., the medial surface). The wear track is 15 mm long, which was designed based on the contact area length on the medial tibial plateau during knee flexion.

**Figure 4:**
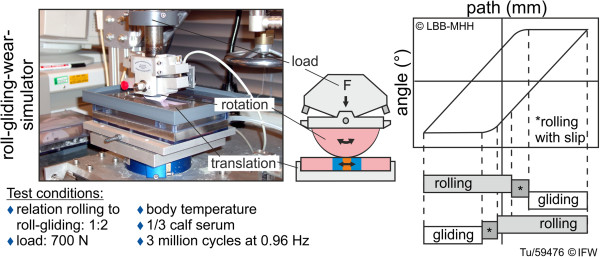
Principle of the rolling-gliding wear simulator.

Three articulation mechanisms of the tibiofemoral joint – pure rolling, rolling-slipping and gliding – are accounted for by the wear simulator. The simplified tibial component (base plate) is oscillated along a horizontal axis by a servo-motor with an adjustable eccentric. The base plate thus rolls and glides against the simplified femoral component (semicylindrical counterbody, radius 32 mm) under axial loading from a dead weight (Figure 4). Adjustable stoppers on the counterbody fixture limit this component’s free rotational range of motion, thus enabling control of the ratio of rolling to gliding. Reproducible positioning of the test pieces is ensured through: first, the use of keyways in the ceramic pieces corresponding to inverse shapes in the stainless steel machine fixtures, for positioning along the translational axis; second, customized plastic spacer blocks for positioning perpendicular to this axis; and third, the ability for the fluid tray to rotate freely about this axis to account for small malalignments of the top and bottom fixtures.

Wear testing was carried out under a constant vertical load of 700 N (+14 N structure weight) on the counterbody. This load corresponds to one half of the mean knee compressive force (i.e., that applied through one of the two tibiofemoral contact areas) calculated over the stance phase of a gait cycle (ISO14243). The ratio of rolling (with or without slip) to a superposition of rolling and gliding was set at 1:2, approximating the physiological articulation in the range of knee flexion associated with the aforementioned stance phase. The wear simulator operates at 1 Hz, and the simplified components are tested while bathed in fetal calf serum diluted to a protein content of 20 g/L, at a temperature of 37 +/− 2°C. Distilled water was regularly added to the serum to compensate for evaporation and thus maintain a consistent protein concentration in the testing medium.

Wear was measured gravimetrically according to ASTM standards F2025 and F1715. The components were cleaned and dried as specified by these standards prior to weighing. After wear testing, these processes were repeated under identical conditions, and gravimetric wear was calculated by the change in mass. Volumetric wear was computed using the known material density. Wear measurements were carried out after 100,000, 500,000, 1 million, 2 million and 3 million cycles. Further details of the wear simulator and the procedures of testing and gravimetric wear assessment have been previously reported [24].

### Topography measurement

**Figure 5:**
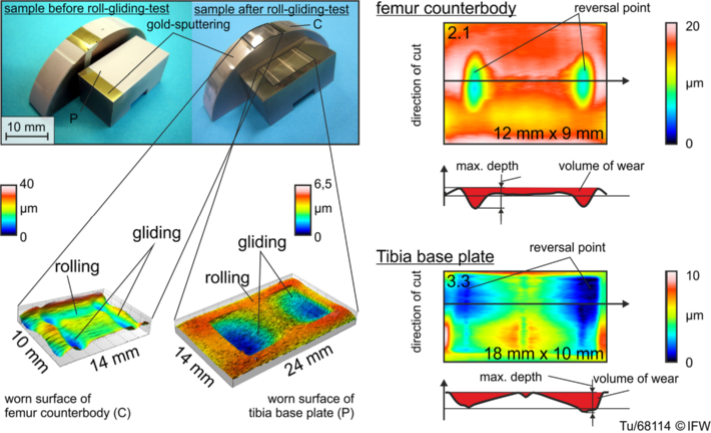
Optical wear and wear depth measurement using laser scanning microscopy.

Two methods were used to measure the topography of the ground and polished surfaces before and the worn surface after wear testing. Firstly, roughness parameters (specifically, Ra, Sa, Rz, and Sz) were measured with a confocal white-light microscope (μsurf®, Nanofocus AG, Oberhausen, Germany) with a measuring field of 160 μm × 160 μm (Figure 5) and a vertical resolution of 0.0015 μm. Secondly, a scanning electron microscopy (SEM) device (EVO 60VP, Carl Zeiss Industrielle Messtechnik GmbH, Oberkochen, Germany), was used to image and evaluate the articulating surfaces at a resolution of 4 nm.

For a second, independent set of wear measurements, wear volume was measured by optical methods following completion of wear testing. For this, a laser profilometer (μscan®, Nanofocus AG) was used, with a measuring range of 200 mm × 200 mm × 1 mm (Figure 5) and a maximum vertical resolution of 0.02 μm. The volume of material removed during the wear tests was calculated to be the difference between the final (worn) surface and the initial surface, i.e., the volume of the ‘crater’. The initial surface was estimated by generation of a polynomial surface that fits over the non-worn areas of the components, using MountainsMap® software (DigitalSurf, Besançon, France).

## Results

### Manufacturing conditioned wear of implant components

**Figure 6:**
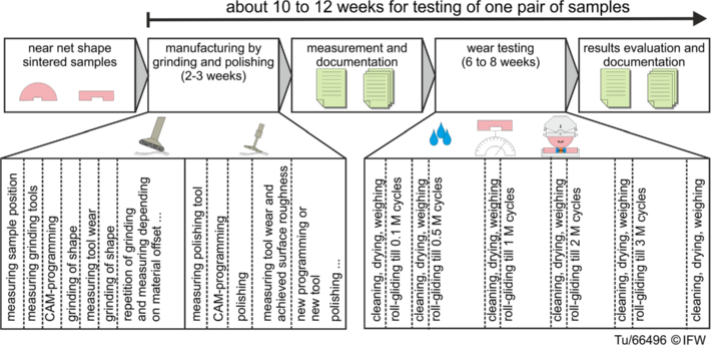
Procedure of manufacturing and wear testing.

The overall procedure for manufacture, wear testing and documentation is shown in Figure 6. Sintered test piece bodies were measured in the aforementioned coordinate measuring machine (CMM), from which CAM-programming of the grinding tool paths took place. Precise measurement of the tool shape was necessary due to a five-axis machining kinematic and complex workpiece geometry. In the grinding step, removal of one material layer of 20 μm depth took approximately 20 minutes, but depended on the type of ceramic and the grinding tool. After grinding, both tool wear and material removal were measured. After the desired shape of a given sample had been achieved, polishing was performed similarly, and took approximately 200 min, with the increase mostly due to smaller tools. After all machining steps had been completed, the geometry of the samples was measured by the CMM, and the surface topography was inspected by optical methods. Wear testing was then able to commence.

This manufacturing procedure took between 2–3 weeks for a single batch of samples, which included cutting tool programming, grinding and polishing, wear compensation, and surface measurement. However, for a hypothetical all-ceramic knee implant component, the complete machining time (i.e., grinding and polishing) would be dependent on the workpiece oversize of the sintered component. Ideally, this oversize would be less than or equal to 150 μm, which would then require one rough grinding step (approximately 20 min), one fine grinding step (20 min) and one polishing step (<200 min, depending on tool size).

### Study design on wear behavior

The specific questions relating to wear behavior (cf. 1) were addressed after samples of the ZTA ceramic had been machined by grinding and polishing (Figures 1 and 2).

**Figure 7:**
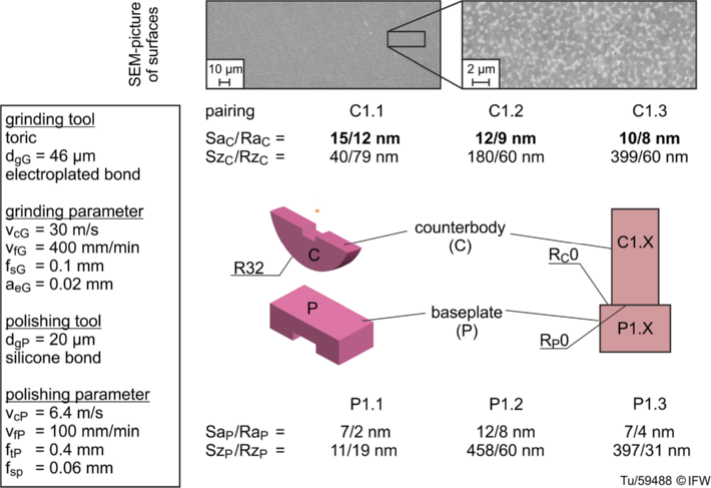
Roughness and geometry of the ground and polished samples (C1.x and P1.x).

To address the first research question – the influence of machining quality - the same machining process was applied to three component pairs and the roughness parameters Sa, Ra, Sz and Rz were measured with a white-light microscope (cf. 2.5). The simplified femoral components (counterbodies) were semi-cylindrical with sagittal plane radii of R = 32 mm, and the simplified tibial components (base plates) were planar. These samples were named C1.x and P1.x. The mean roughness values were: Sa_C_ of 12.33 nm, Ra_C_ of 9.66 nm, Sa_P_ of 8.7 nm and Ra_P_ of 4.7 nm. The SEM images displayed even and ductile-machined surfaces (Figure 7, top). The pores of the ceramic material were closed, and the surface was finished.

**Figure 8:**
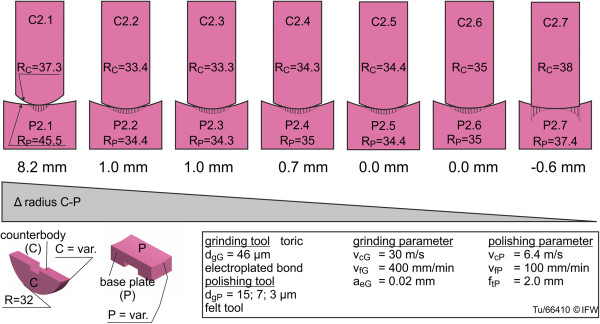
Tested specimen geometries with different levels of frontal plane congruence (C2.x and P2.x).

To begin to address the second research question, the frontal-plane radii of conventional femoral/tibial implant components were measured. An improved load distribution within the implant may be expected with smaller radii differences between the components, while greater radii differences may be advantageous for restoring medio-lateral translation kinematics. To examine wear differences with respect to surface congruence, seven sample pairs with frontal-plane radii differences (base plate radius R_P_ minus counterbody radius R_C_ = 8.2 mm; 1.0 mm, 1.0 mm, 0.7 mm; 0.0 mm, 0.0 mm; -0.6 mm) were examined (Figure 8). The radius in the plane of movement (sagittal plane) remained at R = 32 mm, equal to the previous samples (Figure 7). For the last sample an unfavorable ratio was intentionally used: the radius of the counter body is 0.6 mm larger than that of the base plate, and theoretically this may cause unfavorable edge effects and high stress concentrations when undergoing wear testing. All components were machined with identical process steps to the previous samples. The mean surface roughness values of these sample pairs were: Sa_C_ of 25.7 nm, Ra_C_ of 11.9 nm, Sa_P_ of 44.5 nm and Ra_P_ of 14 nm.

**Figure 9:**
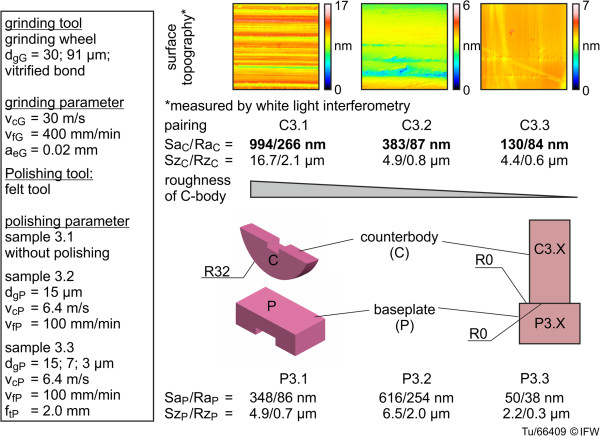
Different roughness and geometry of the ground and polished samples (C3.x and P3.x).

As a pre-investigation, the influence of roughness on wear under knee implant conditions was also determined, but for a small sample size. Three sample pairs with identical geometry to the first three were used, but with varying levels of surface roughness, with Sa values of the counterbodies that ranged from 130 nm to 994 nm (Figure 9). Figure 8 also shows the different topographies of the cylindrical component surfaces. There were clearly recognizable grinding marks on the sample with the roughest surface, C3.1, while samples C3.2 and C3.3 displayed smoother surfaces.

### Results of wear investigation

**Figure 10:**
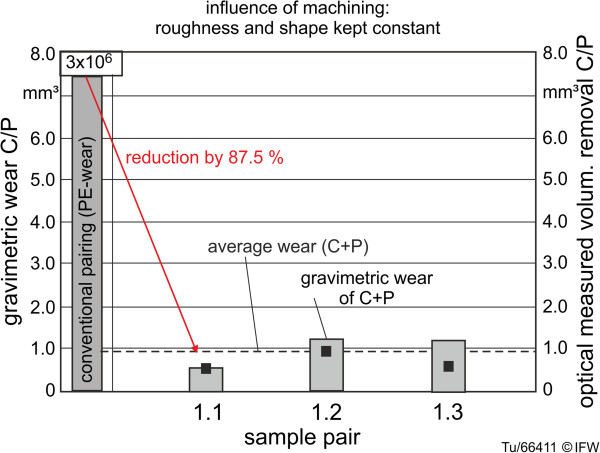
Influence of machining at constant roughness on absolute wear after 3 million cycles.

The wear behavior of the samples throughout the 3 million wear cycles displayed roughly linear wear after a brief “running-in” period of approximately 500,000 cycles. The wear measurements from gravimetric and optical methods were reasonably consistent (Figure 10), with the average wear of the first three pairs (1.1-1.3) differing between methods by around 25% (0.72 mm^3^ optical, 0.96 mm^3^ gravimetric). The wear of the base plates was generally slightly greater than the wear of the counterbodies. In comparison to a conventional implant pairing (CoCr-PE) tested using the same wear simulator and protocol, the ceramic-ceramic pairings showed a reduction of wear behavior of almost 90% (wear of PE component: 7.62 mm^3^ after 3 million cycles).

**Figure 11:**
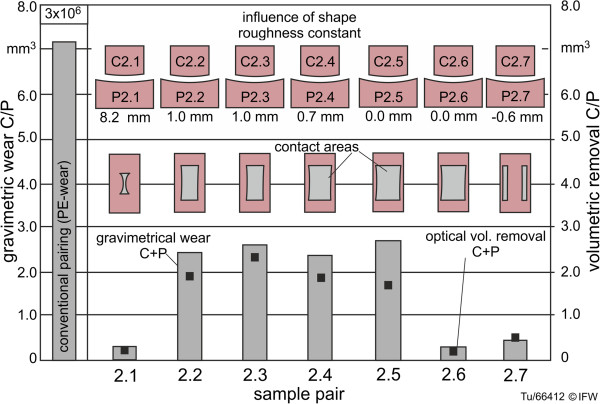
Wear of specimen with different frontal-plane geometry (increasing congruency of contact areas) and constant roughness (C2.x and P2.x).

**Figure 12:**
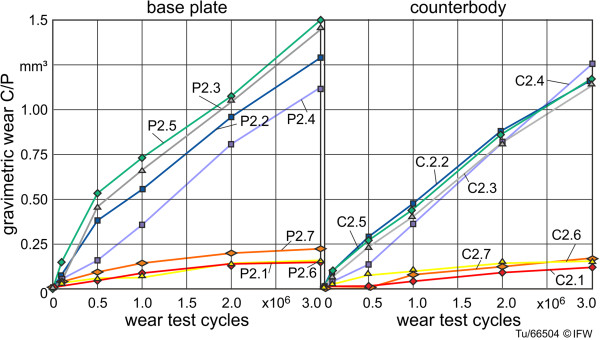
Wear behavior during wear testing of specimen with different frontal plane geometry (increasing congruency of contact areas) and constant roughness (C2.x and P2.x).

For the samples with different frontal-plane radii and associated levels of surface congruence, contact pressure would certainly increase with increased radius difference due to a reduced contact area. However, high-strength ceramic materials are capable of withstanding high pressures, and wear is thought to be predominantly affected by the number of micro contacts [26]. The number of micro contacts is determined by the size of the contact area; thus, wear should increase with increased contact area, and therefore, decreased radius difference. Our results show low wear for frontal-plane radius differences of 8.2 mm, 0 mm and −0.6 mm, but higher wear for radius differences of 0.0 mm, 0.7 mm, 1.0 mm, and 1.0 mm (Figures 11 and 12). The radius differences, as mentioned in section 2.3, are vulnerable to small measurement errors.

The specimens with high levels of congruency (2.2: 1.0 mm, 2.3: 1.0 mm, 2.4: 0.7 mm, 2.5 0.0 mm) showed very similar rates of wear after 3 million wear test cycles. Sample pairs 2.1 (unconforming surfaces, central point load) and 2.7 (unconforming surfaces, peripheral point loads) displayed considerably lower wear than the conforming pairs. Sample pair 2.6 also displayed a low rate of wear, possibly due to the margin of error of the radius measurement, because if the difference in radius was −0.1 rather than 0.0, contact and wear would occur at peripheral point loads.

The results of the pre-investigation of the influence of roughness on wear are shown in Table 1. For all three samples with varying levels of surface roughness (samples 3.1-3.3), the average wear rate was similar to the highly polished samples (1.1-1.3) (Figure 10). The samples with the roughest surface actually produced the lowest wear – 0.43 mm^3^ (1.87 mg) after 3 million cycles, but the variability was high and the sample size was very low, and there was no identifiable relationship.

**Table 1:** Wear of sample with varying roughness

***Combination***	***CoCr-PE***	***Average of group 1.1-1.3***	***C3.1 / P3.1***	***C3.2 / P3.2***	***C3.3 / P3.3***
*Gravimetrical wear [mm*^*3*^*/3x10^6 cycles]*	*7.62*	*0.97*	*0.43*	*0.15*	*0.86*

**Figure 13:**
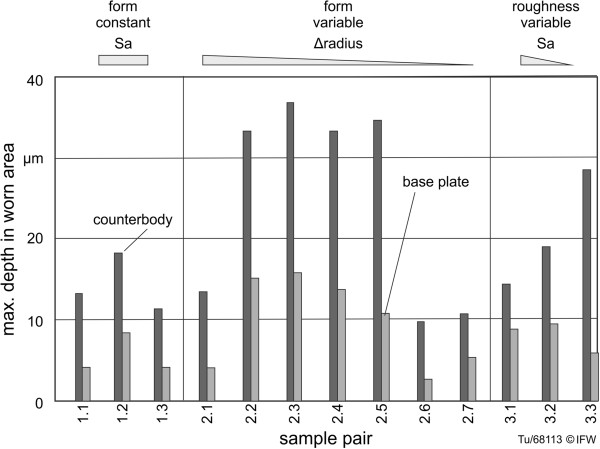
Maximum depth of wear area for all samples 1.1 – 3.3.

An additional analysis of wear behavior involved measurement of the maximum depth of the worn areas (Figures 5 and 13). This was performed with a laser confocal sensor system that cut into the worn surfaces. The base plates showed a “W”-shaped wear depth along the direction of movement (see example in Figure 5). The area of pure rolling can be identified in the center, at which low wear occurred. The areas of rolling and gliding produced the greatest wear depths around the locations of cycle reversal.

Figure 13 illustrates a comparison of the maximum wear depth for all tested specimens. A line contact of high polished surfaces (group 1) achieved maximum depths similar to samples with low congruency (group 2: 2.1, 2.6 and 2.7). For pairings with high surface congruence (3.2a-c and 3.3), greater wear and wear depth were observed when compared with pairings with lower surface congruence. Nevertheless, the wear and wear depth of ceramic samples was considerably lower compared to a conventional material combination.

**Figure 14:**
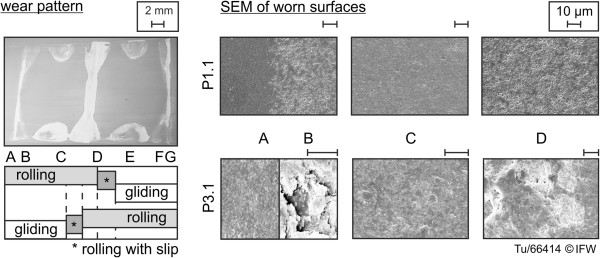
SEM analysis of the worn surfaces of the base plates.

Scanning electron microscopy (SEM) images were used to identify wear mechanisms (Figure 14). The wear pattern of the base plates always contained three lines (Figure 14, labels B, D, and F). Highly polished samples (e.g., P1.1) had uniform wear areas, the polished surfaces were slightly roughened and the pores of the ceramic were opened. In the area of pure rolling (Figure 14, label D), micro-pitting occurred, while in areas of gliding (Figure 14, label C) there was no breakaway of the ceramic surface. Thus, it can be concluded that the material removed was powdery, and caused only by abrasion. The pre-investigation shows that a sample with an initially rougher surface (e.g., P3.1) was mainly worn in areas of pure rolling and rolling with slip. This resulted in micro-chipping and intergranular fracture of the ceramic surface. Abrasion was also observed on the double-curved samples (2.1 to 2.7), similar to the similarly polished surfaces of samples 1.1 to 1.3. In previous studies, pitting and abrasion have been found to depend on wear test kinematics, load, speed and alumina type [26-30]. Intergranular fracture was also reported by Tipper et al. [29].

The counterbodies showed a similar wear mechanism to the base plates. Due to the fixed point of rotation, the edge regions (Figure 5, reversal point) of the transitions between rolling to rolling-gliding were slightly flattened. Abrasion and micro-chipping were found on all the counterbodies. The double-curved samples C2.1-2.7 showed increasing wear areas with decreased frontal-plane radius differences (cf. 3.1.1).

Furthermore, audible “squeaking” noises occurred during testing of the rougher samples. Squeaking is a known phenomenon that occurs in some ceramic hip joints. It is therefore assumed that the effect is led back to the stick slip effect. After a running-in period of 80,000 cycles, the samples 3.1-3.3 (with high roughness of contact surfaces) stopped squeaking. A possible explanation for this is that the high roughness peaks of the surfaces were causing the squeaking, and these were removed during the running-in period.

## Discussion

Analysis of tribological pairings under appropriate loading and kinematic conditions is of great importance for the design and manufacturing strategy for a novel low-wear knee endoprostheses. Two major aspects of such tribological studies are the surface topography and the geometrical tolerances of the implant components [25,31]. The present study found a strong effect of frontal-plane surface conformity on the wear mechanism and volumetric wear rate: increased conformity led to increased wear. A pre-investigation into the effect of surface roughness with a small sample size showed highly variable wear with no clear trend.

The average wear rate of ceramic single-curved samples with identical surface topography was 0.31 mm^3^ per 1 million cycles (Table 2). Previous experiments showed that a geometrically identical specimen of the conventional knee implant material combination (CoCr-PE) displayed wear of the polyethylene component of 2.54 mm^3^ per 1 million cycles. Thus, the wear of a conventional pairing using this simulator was more than eight times the wear of a ceramic pairing.

**Table 2:** Average wear rate reduction by use of ceramic-ceramic material combination

***Combination***	***CoCr-PE***	***Ceramic-ceramic (Single-curved, group 1)***	***Ceramic-ceramic (Double-curved, group 2)***
*Average wear rate [mm*^*3*^*/10^6 cycles]*	*2.54*	*0.31*	*0.53*
*Improvement to CoCr-PE [%]*		*87.5*	*79.1*

Similar results for hip implants were found by Morlock et al. [32], who summarized the findings of wear studies for different material combinations. For metal-PE hip combinations, wear rates between 3–80 mg/10^6 cycles were found. All-ceramic material combinations displayed wear rates between 0.02-0.30 mg/10^6 cycles. Minoda et al. [33] also reported reduced wear by ceramic-PE knee implants relative to CoCr-PE implants.

The wear tests with varying differences in frontal-plane radii showed an increase in wear with increasing contact area (i.e., reduced difference in radii). This can be explained by an increasing number of micro contacts. Similar results for increasing contact stress and decreasing wear rate by increasing the radial clearance have been found for hard-soft material combinations (CoCr-PE) [34-36]. Abdelgaied et al. [34] showed by computational models of PE-inserts in total knee replacements that less conforming geometries had a lower predicted wear under both intermediate and high kinematics. The wear rates for the more conforming inserts were more than three times that for the less conforming insert. Uma et al. [35] demonstrated in PE-inlays that the wear rate increases with the increasing contact area, too. Hereby, the volumetric wear and contact area are a function of the effective radius of the contact geometry. Additionally, Mazzucco et al. [36] showed that the volumetric wear was independent of normal load within the measured range of his study.

Due to the limited number of sample pairs tested, further investigations are needed for a more complete understanding of the influence of frontal-plane radius differences on wear behavior. As this research uses simplified components and simplified rolling-gliding kinematics, the data cannot be directly compared with wear test results from a total knee endoprosthesis simulator. Nevertheless, the results of the simplified samples of cobalt-chromium and PE showed similar behavior to total knee prostheses [23,37].

Further analysis is required to determine if the different topographies of the components leads to reduced wear due to the changing lubrication film on ground surfaces. Furthermore, if the application of a specific structure/pattern on the articulating surfaces has been shown to improve macro lubrication [31,38], and this also warrants further investigation with the materials and testing methods used in the present study.

The results of the pre-investigation did not show any clear relationship or trend relating surface roughness and wear, but the wear rate of rougher specimen was similar to polished samples. While in conventional material combinations (CoCr-PE), a highly polished surface quality displays the lowest wear [39,40], other studies have shown that the effect of roughness is particularly remarkable or negligible if the wear test duration is sufficiently high [40-42]. In this case, rough surfaces tend to be smoothed and smooth surfaces tend to be roughened over the high number of wear cycles. After a certain running-in period, implants have been shown to display similar levels of roughness and similar wear rates [43]. This behavior has also been described in purely tribological studies on both ductile metallic samples [44] and brittle ceramic materials [45-48]. As a result, further investigations are needed to check of a highly accurate polishing of the surface Ra < 20 nm is required, or rough polished or precision ground surface is sufficient for wear and the manufacturing costs for ceramic implants can be reduced.

The wear mechanisms found in the present study relating to surface conformity generally agree with the literature, but are the first to report wear of components made from this commonly used implant material under loading and surface conditions similar to that of a knee joint replacement.

## Conclusions

A process chain for the manufacturing of all-ceramic implants was successfully developed. Surface roughness levels were able to be predicted after grinding by means of calculated and verified models. Therefore, it was possible to determine a process layout in advance. The subsequent polishing step, which levels the roughness peaks, is being advanced in current research with an aim to also successfully predict surface roughness after polishing and to increase productivity.

Using grinding and polishing methods, simplified all-ceramic implant components were manufactured. The influences of surface geometry on implant wear in a rolling-gliding wear simulator were examined. The results showed that it is possible to attain significantly reduced wear rates through the use of all-ceramic implants compared to conventional material combinations such as cobalt-chromium-molybdenum alloys with polyethylene. Increased frontal-plane radius differences (and therefore increased stresses under loading) of the components did not result in breakage or other failure, and additionally displayed reduced wear compared to components with highly congruent surfaces.

Future research will focus on the verification of the wear results, investigation of the effect of surface roughness on wear, grinding with toric grinding pins, polishing of unicondylar-ceramic implant components with resilient diamond tools and subsequent testing of wear and kinematic behavior. Our ultimate aim is to manufacture and test an all-ceramic total knee endoprosthesis.

## Abbreviations

C: Counterbody; CAM: Computer added manufacturing; CoCr: Cobalt-chromium-molybdenum alloy; CMM: Coordinate measurement machine; F: Force in N; HIP: Hot isostatic pressing; P: Base plate; PE: Polyethylene; Ra,Sa: Arithmetic average roughness (2D),(3D); Rz,Sz: Roughness depth (2D), maximum height of surface (3D); SEM: Scanning electron microscope; ZTA: Zirconia toughened alumina oxide.

## Competing interests

The authors declare that they have no competing interests.

## Authors' contributions

AT carried out machining investigations for grinding and polishing processes. She machined all samples and did surface metrology and drafted the manuscript. AT and TC interpreted the results. TC assisted with manuscript writing. CB and CH supervised the wear test machine development and testing. BD and JK advised on machining techniques and discussion of results, and helped with manuscript writing. All authors read and approved the final manuscript. Berna Richter, who developed the wear test machine, was heavily involved in wear testing and study design, but not in drafting the paper.
